# Differentially expression and function of circular RNAs in ovarian cancer stem cells

**DOI:** 10.1186/s13048-022-01014-z

**Published:** 2022-08-17

**Authors:** Eun Jung Sohn

**Affiliations:** Pusan National University, Yangsan, 50612 Republic of Korea

**Keywords:** circRNA, microRNA, Ovarian cancer stem cells,

## Abstract

**Background:**

Circular RNAs (circRNAs) are noncoding RNAs that regulate miRNA expression; however, their functions in cancer stem cells (CSCs) are not well known.

**Methods:**

To determine the function of differentially expression of circRNAs associated with ovarian CSCs, circRNA profiling was conducted using a circRNA-based microarray on sphere-forming cells derived from A2780 and SKOV3 epithelial ovarian cancer cells termed A2780-SP and SKOV3-SP compared to monolayer cells such as A2780 and SKOV3 cells, respectively. Gene Ontology (GO) enrichment and Kyoto Encyclopedia of Genes and Genomes (KEGG) pathway analyses were performed to predict the biological functions of the circRNAs expressed in CSCs.

**Results:**

The circRNA-based microarray data showed that 159 circRNAs were significantly upregulated (fold change > 1.5) and 55 circRNAs were downregulated in ovarian CSCs compared to monolayer cells. GO and KEGG enrichment analysis of differentially expressed circRNAs in ovarian CSCs showed that they were mainly involved in cell cycle, histone modification, cellular protein metabolic process, cell cycle, apoptotic signaling pathway, and ubiquitin-mediated proteolysis in ovarian cancer. In addition, the hsa-circRNA000963-miRNA-mRNA regulatory network was constructed based on potential target of miRNAs. These analyses involved that the biological function of the hsa-circRNA00096/miRNA/mRNA network was involved in signaling pathways regulating pluripotency of stem cells, PI3K-Akt signaling pathway, cell cycle, p53 signaling pathway, Wnt signaling pathway, calcium modulating pathway, and production of miRNAs involved in gene silencing by miRNA.

**Conclusions:**

Our data demonstrate the expression profiles of circRNAs in ovarian CSCs and suggest that circRNAs may be potential diagnostic and predictive biomarkers of ovarian cancer.

**Supplementary Information:**

The online version contains supplementary material available at 10.1186/s13048-022-01014-z.

## Background

Ovarian cancer is an aggressive gynecological cancer that develops chemoresistance and has poor prognosis [1]. Most ovarian cancers are of epithelial origin and are classified as Type I, including endometrioid, low-grade serous, clear-cell, or mucinous carcinomas, and Type II, such as high-grade serous carcinoma displaying chemotherapy resistance and different prognoses [2]. Most ovarian tumors (75%) and ovarian malignancies (90–95%) are epithelial ovarian cancers of different origins. The 5-year survival rate of ovarian cancer depends on the dissemination of the disease at the time of diagnosis [3].

Cancer stem cells (CSCs) or spheroids have stemness properties such as self-renewal, differentiation, and cancer metastasis [4]. In addition, CSCs are resistant to chemotherapy regimens and contribute to the onset of tumor relapses [5]. CSCs are present in various tumor types, such as colon, lung, leukemia, breast, and brain tumors [6–8]. Moreover, signal transduction pathways in CSCs have been suggested as main targets for new therapeutics [9, 10].

Circular RNAs (circRNAs) are noncoding RNAs that exist in many species and are characterized by a covalently closed loop that lacks either 5′–3′ polarity or a poly-adenylated tail [11]. Recent studies have reported that circRNAs play a role in gene expression by acting as miRNA sponges [12]. In addition, circRNAs are involved in stemness regulation, tumor propagation, and cancer metastasis are affected in several diseases such as inflammatory bowel and Alzheimer’s disease [13, 14] and have been suggested as diagnostic or prognostic biomarkers because of their high biological stability [15]. Additionally, circRNAs play an essential role in the onset and progression of ovarian cancers. For instance, hsa-circ-0016347 enhances the proliferation and metastasis of osteosarcoma cells by acting as a sponge of miR-214 [16].

Although several studies have reported on the function of circRNAs in ovarian cancer, the expression profiles and potential roles of circRNAs in ovarian CSCs have not been investigated. To our knowledge, this is the first study to report the presence of 214 differentially expressed circRNAs in ovarian CSCs compared to monolayer cells, providing a novel insight into the underlying mechanism of ovarian CSCs.

## Results

### circRNA expression profiles in ovarian CSCs

**Fig. 1:**
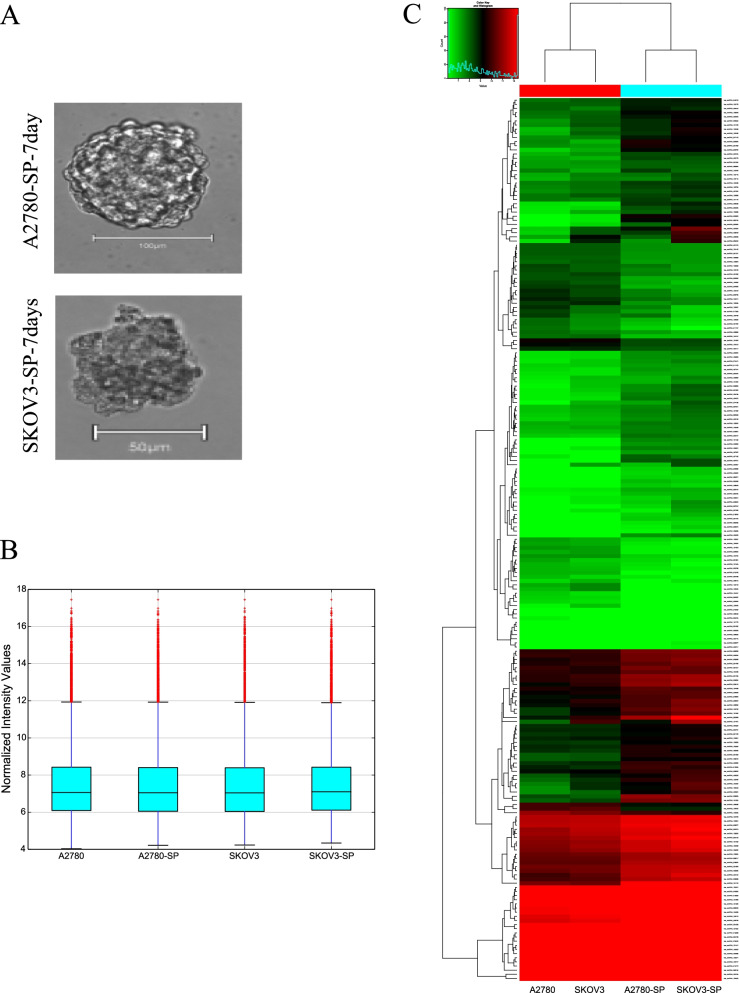
Differentially expression of circRNAs in ovarian cancer stem cells. **A** A representative image of ovarian cancer stem cell sphere formation originating from A2780 (upper) and SKOV3 (lower) cells on day 7 of culture. **B** The box plot shows variations in circRNA expression in ovarian cancer stem cells derived from A2780 and SKOV3 cells compared to control. **C** Heat map of the circRNA microarray profiles representing the expression of circRNAs between the adherent cells (A2780, SKOV3) and cancer stem cells (A 2780-SP, SKOV3-SP). Green color showing lower expression levels and red color indicating higher expression levels

**Fig. 2:**
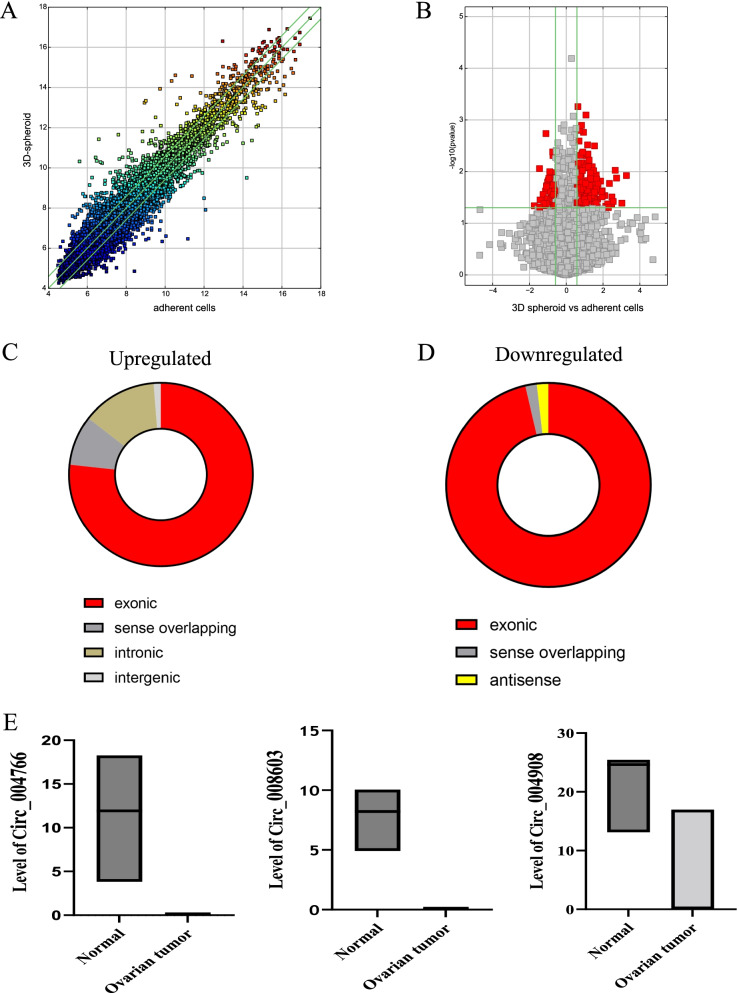
Distributions of circRNAs in human chromosomes in ovarian cancer stem cells. **A** Scatter plots representing differentially expressed circRNAs in ovarian cancer stem cells. circRNAs above and below the border green line show more than twofold change in expression. **B** The volcano plots representing differentially expressed circRNAs between groups. The green vertical line marks twofold changes, while the horizontal line represents a *P*-value of 0.05. Distribution of differentially upregulated (**C**) and downregulated **D** circRNAs between ovarian cancer stem cells and monolayer cells in human chromosomes. The ratio of circRNAs originated from exonic, intronic, and intergenic regions are shown. **E** The circRNA_004766, circRNA_008603, circRNA_004908 level of the GSE192410 dataset, which contains information on ovarian cancer tumour tissue

In this study, sphere-forming cells derived from A2780 and SKOV3 epithelial ovarian cancer cells, termed A2780-SP and SKOV3-SP cells, respectively, were used as ovarian CSCs to evaluate the role of circRNAs in ovarian CSCs. As shown in Fig. 1A, the spheres were cultured for 7 days, and then images showing morphology of spheres were taken under the phase contrast microscope*.* To identify the expression of circRNAs in ovarian CSCs, microarrays based on circRNAs were conducted on ovarian CSCs (A2789-SP, SKOV3-SP) compared to monolayer cells (A2780 and SKOV3 cells). The box plot represents that the median intensity values in ovarian CSCs (A2780-SP, SKOV3-SP) and monolayer cells (A2780, and SKOV3) were almost similar after normalization (Fig. 1B). As shown in Fig. 1C, hierarchical clustering demonstrates the expression of multiple circRNAs in the CSCs (A2789-SP and SKOV3-SP) and monolayer cells (A2780 and SKOV3 cells). The scatter plot and volcano in Fig. 2A and B shows the differences in circRNA expression between CSCs and monolayer cells. Our results showed that 159 circRNAs were significantly upregulated and 55 circRNAs were significantly downregulated in ovarian CSCs compared with the monolayer cells (fold change > 1.5) (Supplementary material). In A2780-SP cells, 2447 circRNAs were upregulated and 2346 circRNAs were downregulated compared to A2780 (log2 (fold change)| > 1.5 and a *P*-value < 0.05 were used to evaluate significant differences in the expression of circRNAs between the two groups) (Supplementary material). In SKOV3-SP cells, 2447 circRNAs were upregulated and 22,982 circRNAs were downregulated compared to SKOV3 cells (Supplementary material). The top 10 upregulated or downregulated circRNAs in ovarian CSCs compared to monolayers are listed in Table 1. In addition, Circ_004766, Circ_008603, Circ_004908 were downregulated in ovarian CSCs compared to control (supplementary material). Consistent with our data, these circRNAs were also downregulated in the ovarian cancer tumor tissues compared to normal ovary tissues using public dataset (GSE192410) (Fig. 2E).

**Table 1:** The top 10 upregulated and downregulated circRNAs from ovarian cancer stem cells compared to monolayer cells

circRNA	Gene symbol	Fold change	Difference	Pvalue
hsa_circRNA_000963	BCLAF1	9.654822	up	0.011943959
hsa_circRNA_063745	FBLN1	8.1403078	up	0.040880464
hsa_circRNA_008805	ARHGAP23	6.6659578	up	0.013367646
hsa_circRNA_000320	AHNAK	6.3320878	up	0.009441872
hsa_circRNA_101419	STON2	5.8724314	up	0.037875898
hsa_circRNA_101205	AACS	5.6013188	up	0.03108705
hsa_circRNA_092388	INCENP	5.5566526	up	0.04236629
hsa_circRNA_404594	UBQLN4	5.0965895	up	0.016952911
hsa_circRNA_003794	RRM2B	4.8938952	up	0.049647141
hsa_circRNA_405038	ATP2B1	4.8198654	up	0.04364535
hsa_circRNA_100,545	SFMBT2	3.3372101	down	0.045989835
hsa_circRNA_103,418	EPHA3	2.7379827	down	0.008751358
hsa_circRNA_100,542	SFMBT2	2.7134271	down	0.048906928
hsa_circRNA_004908	UBE3D	2.6831921	down	0.035520311
hsa_circRNA_100,484	PCNXL2	2.3906136	down	0.027729139
hsa_circRNA_011167	EPB41	2.3571065	down	0.032371803
hsa_circRNA_104,290	EIF3B	2.3226174	down	0.041535724
hsa_circRNA_100,085	EIF4G3	2.2426993	down	0.035612415
hsa_circRNA_103,597	TBC1D14	2.2176623	down	0.045906881
hsa_circRNA_100,518	ZMYND11	2.2026407	down	0.047528208

### Expression of circRNAs in human chromosomes

Among all differentially expressed circRNAs, 159 upregulated and 55 downregulated circRNAs overlapped between A2780-SP and SKOV3-SP cells. Of the 159 upregulated circRNAs, 122 (76.7%) were transcribed from the exonic region, 14 (8.8%) from the sense-overlapping region, 21 (13.2%) from the intronic region, and 2 (1.2%) from the intergenic region (Fig. 2C), whereas of the 55 downregulated circRNAs, 53 (96.3%) were transcribed from the exonic region, 1 from sense-overlapping region (1.8%), and 1 from the antisense region (1.8%) (Fig. 2D).

### GO functional annotation and pathway enrichment analysis of differentially expressed circRNAs

**Fig. 3:**
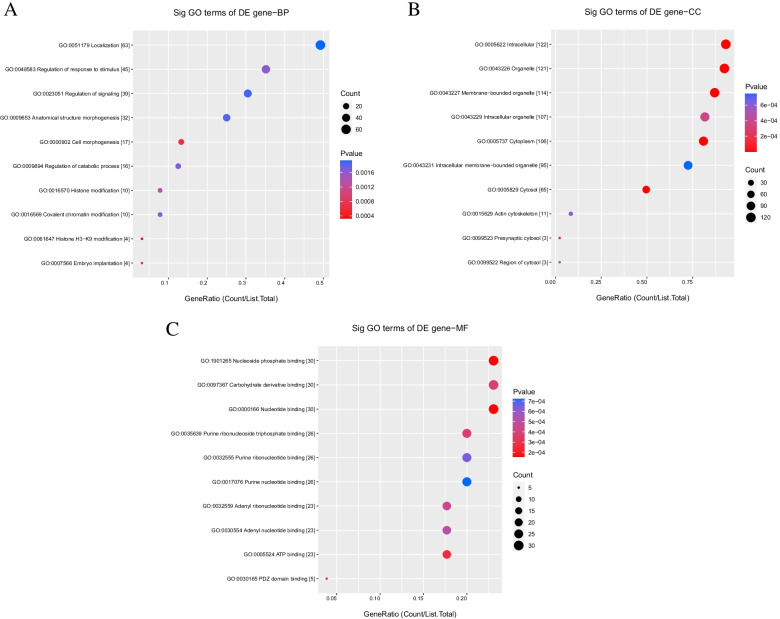
GO analysis for upregulated circRNAs in ovarian cancer stem cells (A2780-SP, SKOV3-SP) compared to monolayer cells. **A** for BP; (**B**) for CC (**C**) for MF. BP: biological process; CC: cellular component; MF: molecular function

circRNAs are encoded by their parental genes, and one of their primary functions is to regulate parental gene expression [17]. To identify the function of circRNAs in ovarian CSCs, we performed GO functional pathway enrichment analysis of parent genes of differentially expressed circRNAs. The 10 most significant GO terms of the upregulated circRNAs in ovarian CSCs are listed in Fig. 3. The identified BP terms were localization, regulation of response to stimulus, regulation of signaling, anatomical structure morphogenesis, cell morphogenesis, regulation of catabolic process, histone modification, covalent chromatin modification, and histone H3-K9 modification (Fig. 3A). The identified MF terms were nucleoside phosphatase binding, carbohydrate derivative binding, nucleotide binding, purine ribonucleoside triphosphate binding, purine ribonucleotide binding, purine nucleotide binding, adenyl ribonucleotide binding, adenyl nucleotide binding, ATP binding, and PDZ domain binding (Fig. 3B). The identified CC terms were intracellular, organelle, membrane-bounded organelle, intracellular organelle, cytoplasm, intracellular membrane-bounded organelle, cytosol, and actin cytoskeleton (Fig. 3C).

**Fig. 4:**
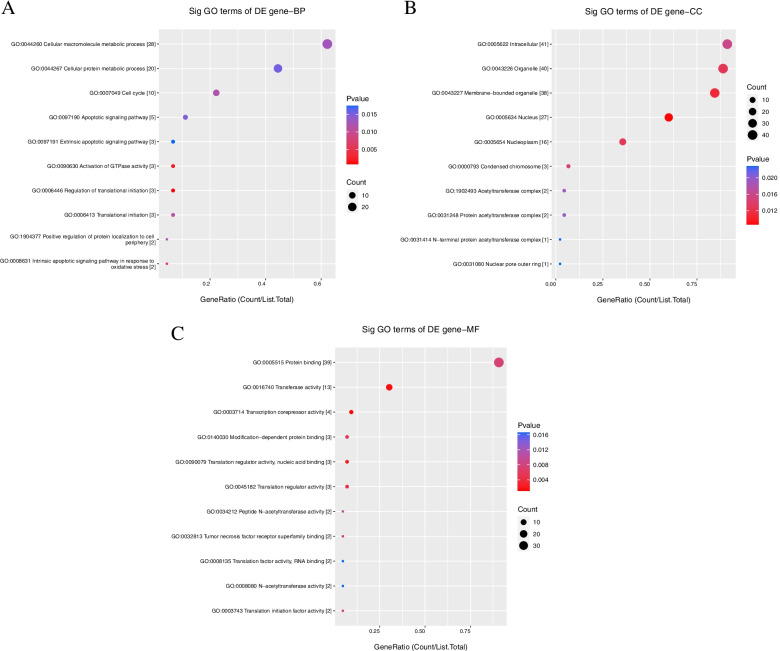
GO analysis for downregulated circRNAs in ovarian cancer stem cells (A2780-SP, SKOV3-SP) compared to monolayer cells (A2780, SKOV3). **A** for BP; (**B**) for CC; (**C**) for MF. BP: biological process; CC: cellular component; MF: molecular function

The 10 most significant GOs of the downregulated circRNAs are listed in Fig. 4. The identified BP terms were cellular macromolecule metabolic process, cellular protein metabolic process, cell cycle, apoptotic signaling pathway, extrinsic apoptotic signaling pathway, and activation (Fig. 4A). The MF terms were protein binding, transferase activity, transcription corepressor activity, modification-dependent binding, translation regulator activity, nucleic acid binding, translational regulator activity, peptide N-acetyltransferase activity, tumor necrosis factor receptor superfamily binding, translation factor activity, RNA binding, N-acetyltransferase activity, and translation initiation factor activity (Fig. 4B). The identified CC terms were intracellular, organelle, membrane-bounded organelle, nucleus, nucleoplasm, condensed chromosome, acetyltransferase complex, protein acetyltransferase complex, N-terminal protein acetyltransferase complex, and nuclear pore outer ring (Fig. 4C).

**Fig. 5:**
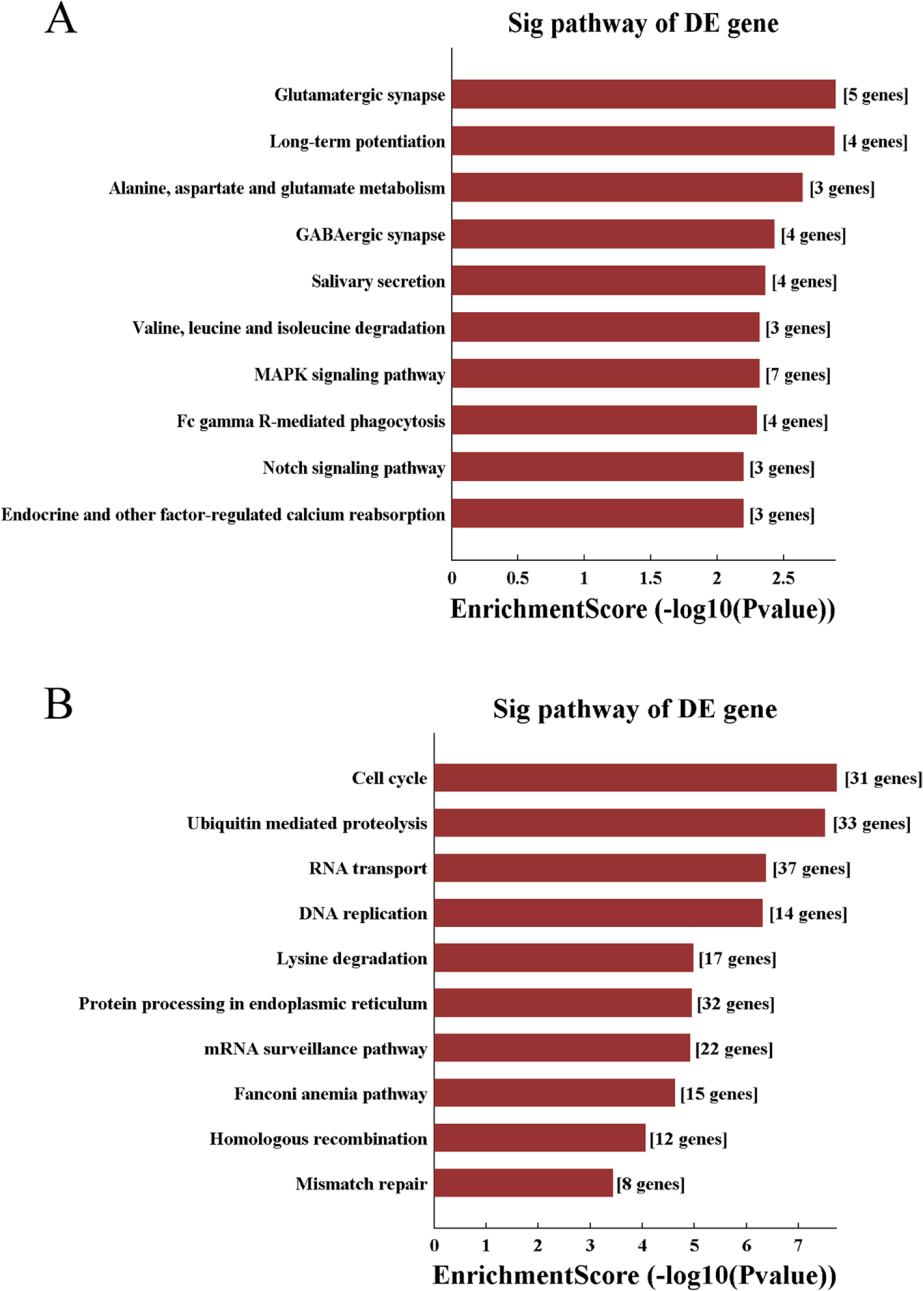
KEGG pathway analysis for upregulated or downregulated circRNAs in ovarian cancer stem cells (A2780-SP, SKOV3-SP) compared to monolayer cells (A2780, SKOV3). KEGG pathway analysis shows the top 10 enriched pathways for upregulated **A** and downregulated (**B**) circRNAs in the ovarian cancer stem cells

Next, to investigate the functional roles of circRNAs in CSCs, the KEGG pathway was used with mRNAs transcribed from the parent genes of differentially expressed circRNAs in ovarian CSCs. From the upregulated and downregulated circRNAs in ovarian CSCs, the 10 most significant KEGG pathways are listed in Fig. 5 A and B. As shown in Fig. 5A, KEGG analysis of upregulated circRNAs indicated enrichment in glutamatergic synapse; long-term potentiation; alanine, aspartate, and glutamate metabolism; GABAergic synapse; salivary secretion; valine, leucine, and isoleucine degradation; MAPK signaling pathway; Fc gamma R-mediated phagocytosis; Notch signaling pathway; and endocrine and other factor-regulated calcium reabsorption. KEGG analysis of downregulated circRNAs revealed enrichment in cell cycle, ubiquitin-mediated proteolysis, RNA transport, DNA replication, lysine degradation, protein processing in endoplasmic reticulum, mRNA surveillance pathway, fanconi anemia pathway, homologous recombination, and mismatch repair (Fig. 5B).

### Survival analysis of parental gene expression in ovarian cancer cells

**Fig. 6:**
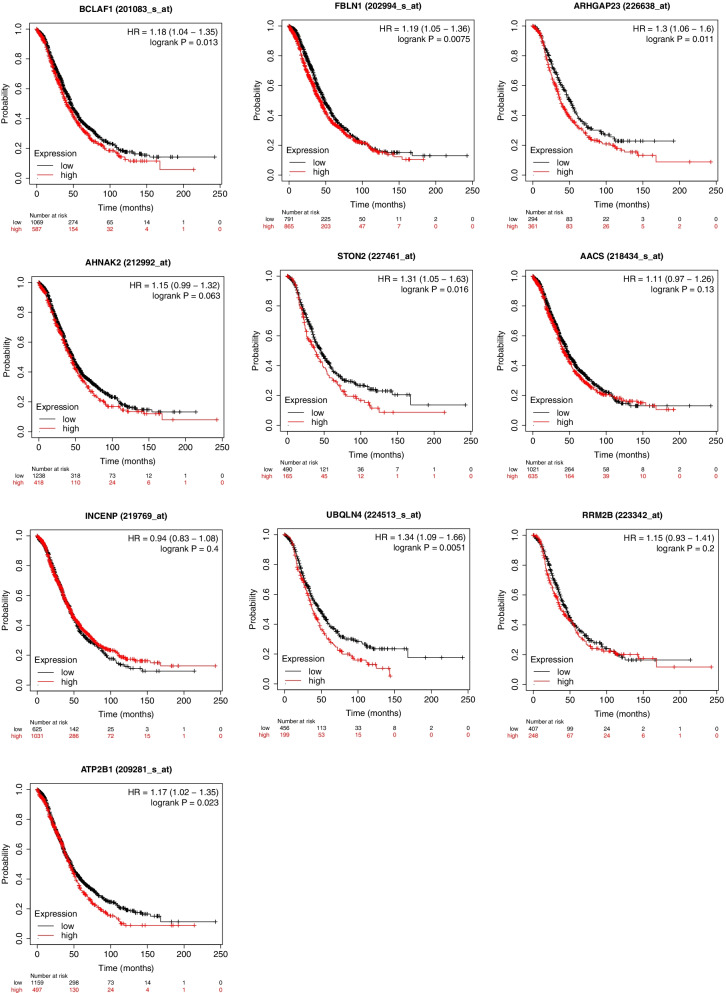
Survival analysis of expression of parental genes (*BCLAF1, FBLN1, ARHGAP23, AHNAK2, STON2, AACS, INCENP, UBQLN4, RRM2B, ATP2B1*) in ovarian cancer cells

circRNAs are encoded by their parental genes, and one of the primary functions of circRNAs is to regulate parental gene expression [17]. To understand whether these parental genes are related to ovarian cancer, the association between the expression of the parental genes of the top 10 circRNAs and survival was examined. We found that the expression of six genes (*BCLAF1*, *FBLN1*, *ARHGAP23*, *STON2*, *UBQLN4*, and *ATP2B1*) had a significant positive correlation with the survival rate of patients with ovarian cancer (Fig. 6).

### Prediction of common MREs of differentially expressed circRNAs and mRNAs

It has been reported that circRNAs can sequester relevant miRNAs through MREs to post-transcriptionally regulate gene expression [18]. Therefore, we investigated potential miRNA targets of the validated circRNAs using miRNA target prediction software. The putative MREs of differentially expressed top 10 upregulated and downregulated circRNAs are listed in Tables 2 and 3.

**Table 2:** Prediction of miRNA binding sites on the top 10 upregulated circular RNAs

circRNA	Potential miRNA targets (No. MREs)				
	MRE1	MRE2	MRE3	MRE4	MRE5
hsa_circRNA000963	hsa-miR-629-3p	hsa-miR-298	hsa-miR-424-5p	hsa-miR-497-5p	hsa-miR-15b-5p
hsa_circRNA063745	hsa-miR-153-5p	hsa-miR-342-3p	hsa-miR-660-3p	hsa-miR-489-3p	hsa-miR-545-3p
hsa_circRNA008805	hsa-miR-582-3p	hsa-miR-103a-3p	hsa-miR-107	hsa-miR-188-3p	hsa-miR-135b-5p
hsa_circRNA000320	hsa-miR-515-5p	hsa-miR-519e-5p	hsa-miR-708-3p	hsa-miR-5197-3p	hsa-miR-9-5p
hsa_circRNA101419	hsa-miR-337-5p	hsa-miR-224-3p	hsa-miR-20b-5p	hsa-miR-127-5p	hsa-miR-146a-3p
hsa_circRNA101205	hsa-miR-6868-3p	hsa-miR-4753-3p	hsa-miR-1238-3p	hsa-miR-130b-5p	hsa-miR-6754-3p
hsa_circRNA092388	hsa-miR-146a-3p	hsa-let-7b-5p	hsa-miR-188-5p	hsa-miR-515-3p	hsa-miR-519e-3p
hsa_circRNA404594	hsa-miR-23b-5p	hsa-miR-93-3p	hsa-miR-10b-3p	hsa-miR-23a-5p	hsa-miR-581
hsa_circRNA003794	hsa-miR-639	hsa-miR-885-3p	hsa-miR-138-5p	hsa-miR-572	hsa-miR-412-5p
hsa_circRNA100518	hsa-miR-216a-5p	hsa-miR-208a-5p	hsa-miR-889-5p	hsa-miR-370-5p	hsa-miR-221-5p

**Table 3:** Prediction of miRNA binding sites on the top 10 downregulated circular RNAs

circRNA	Potential miRNA targets (No. MREs)				
	MRE1	MRE2	MRE3	MRE4	MRE5
hsa_circRNA 100,545	hsa-miR-629-3p	hsa-miR-298	hsa-miR-424-5p	hsa-miR-497-5p	hsa-miR-15b-5p
hsa_circRNA 103,418	hsa-miR-153-5p	hsa-miR-342-3p	hsa-miR-660-3p	hsa-miR-489-3p	hsa-miR-545-3p
hsa_circRNA 100,542	hsa-miR-582-3p	hsa-miR-103a-3p	hsa-miR-107	hsa-miR-188-3p	hsa-miR-135b-5p
hsa_circRNA 004908	hsa-miR-515-5p	hsa-miR-519e-5p	hsa-miR-708-3p	hsa-miR-5197-3p	hsa-miR-9-5p
hsa_circRNA 100,484	hsa-miR-337-5p	hsa-miR-224-3p	hsa-miR-20b-5p	hsa-miR-127-5p	hsa-miR-146a-3p
hsa_circRNA 011167	hsa-miR-6868-3p	hsa-miR-4753-3p	hsa-miR-1238-3p	hsa-miR-130b-5p	hsa-miR-6754-3p
hsa_circRNA 104,290	hsa-miR-146a-3p	hsa-let-7b-5p	hsa-miR-188-5p	hsa-miR-515-3p	hsa-miR-519e-3p
hsa_circRNA 100,085	hsa-miR-23b-5p	hsa-miR-93-3p	hsa-miR-10b-3p	hsa-miR-23a-5p	hsa-miR-581
hsa_circRNA 103,597	hsa-miR-639	hsa-miR-885-3p	hsa-miR-138-5p	hsa-miR-572	hsa-miR-412-5p
hsa_circRNA 100,518	hsa-miR-216a-5p	hsa-miR-208a-5p	hsa-miR-889-5p	hsa-miR-370-5p	hsa-miR-221-5p

### Bioinformatics analysis of validated circRNA-miRNA-mRNA networks

**Fig. 7:**
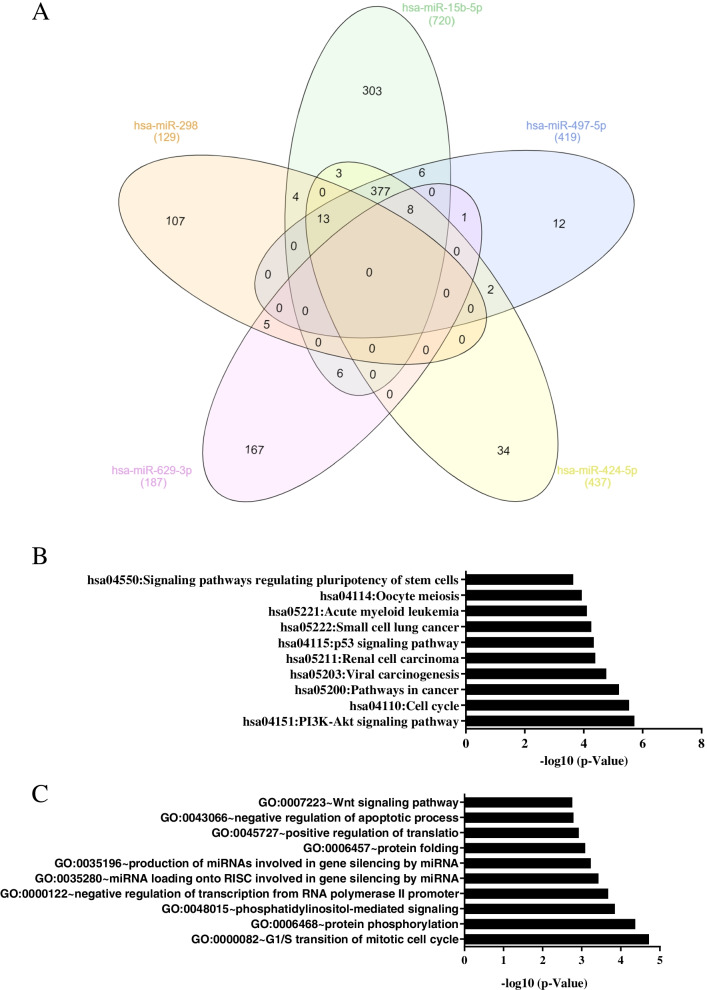
GO and KEGG pathway analysis for the mRNAs in the circRNA-miRNA-mRNA network. **A** A Venn diagram was drawn to identify overlapping targets among five miRNAs (hsa-miR-629-3p, hsa-miR-298, hsa-miR424-5p, hsa-miR-497-5p, and hsa-miR-15b-5p) which possess MRE binding site for hsa_circRNA_000963. KEGG pathways (**B**) and GO terms (**C**) for the target genes are shown. Targets from at least 2 overlapped miRNAs among 5 miRNAs were used for bioinformatics analyses

To examine circRNA-miRNA-mRNA networks, we chose hsa-circRNA000963, which is highly expressed in ovarian CSCs. hsa-circRNA 000963 has five potential miRNA targets (hsa-miR-629-3p, hsa-miR-298, hsa-miR-424-5p, hsa-miR-497-5p, and hsa-miR15b-5p). The miRNA walk database was used to predict miRNA targets. As shown in Fig. 7A, a Venn diagram was drawn to identify overlapping targets among five miRNAs (hsa-miR-629-3p, hsa-miR-298, hsa-miR424-5p, hsa-miR-497-5p, and hsa-miR-15b-5p). To determine the function of the target, we selected the target from at least two overlapped miRNAs among the five miRNAs that were used for bioinformatics analyses. KEGG pathway analysis for 425 target miRNAs showed that they were involved in hsa04550: signaling pathways regulating pluripotency of stem cells, hsa04151: PI3K-Akt signaling pathway, hsa04110: cell cycle, hsa04114: oocyte meiosis, hsa04115: p53 signaling pathway, and so on (Fig. 7B). GO enrichment analysis showed that GO terms were revealed in GO:0007223 ~ Wnt signaling pathway, calcium modulating pathway, GO:0035196 ~ production of miRNAs involved in gene silencing by miRNA, GO:0035280 ~ miRNA loading onto RISC involved in gene silencing by miRNA, GO:0000122 ~ negative regulation of transcription from RNA polymerase II promoter, GO:0000082 ~ G1/S transition of mitotic cell cycle (Fig. 7C).

## Discussion

The treatment of ovarian cancer is difficult because of the high recurrence of the disease and more complicated because of acquired chemoresistance. Cancer stem cell theory suggests that tumour development and progression are guided by undifferentiated stem cells capable of regeneration and tumour initiation [19, 20]. Successful treatment of ovarian cancer is dependent on the eradication of ovarian CSC, as CSCs are the driving force behind the manifestation, progression and recurrence of the disease through conventional treatment [21]. CSCs affect disease recurrence by resisting conventional treatments, such as chemotherapy and radiotherapy [22, 23]. Ovarian CSCs are involved in disease relapse, cancer development, and chemoresistance [24]. Therefore, to develop new therapeutic strategies for ovarian carcinoma, it is important to characterize the molecular phenotype of CSCs. Thus, identifying CSC-specific molecular phenotypes may provide novel therapeutic targets and biomarkers [23]. circRNAs have gained increasing attention as diagnostic markers for various diseases [25–27]. circRNAs are also involved in the development and progression of cancers [28, 29] and regulate CSC functions such as migration, invasion, and self-renewal [29]. In this study, we determined circRNA expression in ovarian CSCs in comparison to that in monolayer cells.

Several studies have shown that circRNAs are associated with the CSC phenotype, such as self-renewal. For instance, circGprc5a is highly expressed in bladder cancer and bladder CSCs, and highly expressed circGprc5a is associated with worse prognosis of patients with bladder cancer. circGprc5a was involved in metastasis and self-renewal of bladder CSCs [29, 30]. In this study, we for the first time performed analysis of circRNA expression in ovarian CSCs (A2780-SP and SKOV3-SP) through circRNA microarray analysis. Our results showed that 159 circRNAs were upregulated and 55 were downregulated in ovarian CSCs (A2780-SP and SKOV3-SP) compared to monolayer cells (A2780 and SKOV3). Our results provide a novel insight into the expression profile of circRNAs involved in possible role in ovarian CSCs.

There is evidence that circRNAs regulate the functions of their parental genes [17]. Therefore, to determine the potential functions of circRNAs differentially expressed in ovarian cancer, the functions of their parent genes were analyzed. GO and KEGG pathway analyses revealed that the parent genes are involved in certain pathways associated with cancer, such as cell cycle, ubiquitin-mediated proteolysis, DNA replication, glutamatergic synapse, and MAPK signaling pathway. Previous studies have shown that CSCs are related to the cell cycle [31] and that MAPK signaling regulates cellular differentiation, proliferation, and survival in cancer [32]. In this study, we also showed that parent genes such as *BCLAF1, FBLN1, ARHGAP23, STON2, UBQLN4*, and *ATP2B1* were significantly positively correlated with the survival rate of patients with ovarian cancer. Thus, our data imply that circRNA may be an important regulator of ovarian cancer.

CircRNAs are rich in miRNA-binding sites (MREs) and act as miRNA sponges. circRNAs regulate disease-associated miRNAs [33]. The circRNAs function as “miRNA sponge” on miRNA signaling which regulate CSC properties [18, 29]. In addition, due to the tumorigenic and invasive activities of CSCs, circRNAs predominantly exert their functions in CSCs [34]. In this study, circRNA 000963 was found to be upregulated in ovarian CSCs, and miRNA candidates (hsa-miR-629-3p, hsa-miR-298, hsa-miR424-5p, hsa-miR-497-5p, and hsa-miR-15b-5p) for circRNA 000963 were identified. The KEGG pathway and GO enrichment analyses of the predicted targets of the miRNAs (hsa-miR-629-3p, hsa-miR-298, hsa-miR424-5p, hsa-miR-497-5p, and hsa-miR-15b-5p) showed that they were enriched in signaling pathways regulating pluripotency of stem cells, cell cycle, p53 signaling pathway, PI3K-Akt signaling pathway, Wnt signaling pathway, calcium modulating pathway, G1/S transition of mitotic cell cycle, and miRNA loading onto RNA-induced silencing complex (RISC) involved in gene silencing by miRNA. It has been reported that p53 signaling is involved in self-renewal and differentiation [35]. Wnt signaling is related to stem cell populations in many malignancies [36] and the PI3K-ATK signaling pathway is involved in the maintenance of spheroid-forming cells [37].

## Conclusions

In conclusion, our data suggest that circRNA deregulation is involved in ovarian cancer pathogenesis. Furthermore, bioinformatics analyses predicted the potential functions of these circRNAs, as well as several potential circRNA-miRNA-mRNA interaction-regulating networks in ovarian CSCs. Additionally, our results indicate potential biomarkers for ovarian cancer and provide functional and mechanistic information on these circRNAs in ovarian CSCs.

## Methods

### Cell culture

Ovarian cancer cells A2780 and SKOV3 were purchased from American Type Culture Collection (ATCC, Rockville, MD, USA) and cultured in RPMI 1640 medium (Thermo Scientific, Waltham, MA, USA) supplemented with 10% fetal bovine serum (FBS, Thermo Scientific, Waltham, MA, USA) and penicillin/streptomycin (Invitrogen Life Technologies, Carlsbad, CA, USA). and maintained at 37 °C in a humidified atmosphere with 5% CO_2_. Sphere-forming cells, CSCs, were prepared from A2780 and SKOV3 cells, as previously described [38]. In brief, for the isolation of sphere-forming cells, A2780 and SKOV3 cells were detached with trypsin/EDTA solution medium (Thermo Scientific, Waltham, MA, USA) and seeded in CSC culture medium containing with Neurobasal™ medium (Thermo Scientific, Waltham, MA, USA) supplemented with 20 ng/ ml basic fibroblast growth Factor (bFGF, R&D Systems, Minneapolis, MN,USA), 10 ng /ml epidermal growth factor (EGF, R&D Systems, Minneapolis, MN,USA), penicillin/ streptomycin (Invitrogen Life Technologies, Carlsbad, CA, USA), and HEPES (Sigma, St Louis, MO, USA), Glutamax (Thermo Scientific, Waltham, MA, USA), B-27 (Thermo Scientific, Waltham, MA, USA) on ultra-low-attachment culture 100 mm^2^ plates (Corning Inc., Corning, NY, USA).

### RNA extraction and quality control

Total RNA from monolayer cells (A2780 and SKOV3) and sphere-forming cells (A2780-SP and SKOV3-SP), cultured for 7 days in sphere-forming conditions, were using the TRIzol reagent (Invitrogen, Carlsbad, CA, USA) according to the standard protocol. Yield and purity were assayed using a NanoDrop ND-1000 (Thermo Fisher Scientific, Wilmington, DE, USA).

### circRNA microarray analysis

Arraystar human circRNA array (Arraystar, Rockville, Maryland, USA) was performed using total RNA from monolayer cells (A2780 and SKOV3) and CSCs (A2780-SP and SKOV3-SP) according to Arraystar’s standard protocols (Arraystar, Rockville, MD, USA). Three samples were pooled for each cell line. circRNAs were considered significantly different when fold change was > 1.5 and the *P*-value < 0.05**.** Heatmap plots were obtained using the R package “heatmap” for the target genes**.**

### Prediction of circRNA-miRNA-target gene interactions

The circBase database and Cancer-Specific CircRNA database (CSCD, https://gb.whu.edu.cn/CSCD/) were used to examine the potential miRNA-binding sites (miRNA response elements, MREs) of circRNAs. circBase provides the chromosomal location and length of the circRNAs. To determine the potential functions of circRNAs in ovarian CSCs, their parental genes were analyzed using DAVID (https://david.ncifcrf.gov/). Biological process (BP), cellular component (CC), and molecular function (MF) were determined using Gene Ontology (GO). The Kyoto Encyclopedia of Genes and Genomes (KEGG) enrichment analysis was used to identify the parental genes of differentially expressed circRNAs in ovarian CSCs and the enriched pathways.

### Gene expression omnibus data set analysis

Gene Expression Omnibus **(**GEO) dataset GSE192410, including ovarian cancer tissues (*n* = 3) and normal ovarian tissues (*n* = 3) was analysed to see differently circRNAs. GEO2R (http://www.ncbi.nlm.nih.gov/geo/geo2r/) was used to identify differentially expressed circRNAs in the ovarian cancer tissues.

### Survival analysis

An online database (http://kmplot.com/analysis/) was used to determine the relationship between gene expression and overall survival (OS) in ovarian cancer cells. Clinical properties of the ovarian cancer patients are as follows; Histology: Serous (n-1232), Endometrioid (*n* = 62), all Stages(1, 1 + 2, 2, 2 + 3, 2 + 3 + 4, 3, 4, 3 + 4), all grades (1, 2, 1 + 2, 2 + 3, 3, 4), TP53 mutation: wild type, mutated [39].

## Supplementary Information

**Additional file 1.**

## Data Availability

The datasets are available from supplementary material in this study. The datasets GSE192410 for this study can be found in the GEO database (https://www.ncbi.nlm.nih.gov/geo/) and are available in the NCBI-GEO repository https://www.ncbi.nlm.nih.gov/geo/query/acc.cgi?acc=GSE192410.

## Abbreviations

GO: Gene Ontology
CircRNA: Circular RNAs
CSC: Cancer stem cells
KEGG: Kyoto Encyclopedia of Genes and Genome
MRE: miRNA-binding sites
